# Fluorination Enables Tunable Molecular Interaction and Photovoltaic Performance in Non-Fullerene Solar Cells Based on Ester-Substituted Polythiophene

**DOI:** 10.3389/fchem.2021.687996

**Published:** 2021-05-10

**Authors:** Ziqi Liang, Mengyuan Gao, Bo Zhang, Junjiang Wu, Zhongxiang Peng, Miaomiao Li, Long Ye, Yanhou Geng

**Affiliations:** ^1^ School of Materials Science and Engineering, Tianjin University, Tianjin, China; ^2^ Tianjin Key Laboratory of Molecular Optoelectronic Science, Tianjin University, Tianjin, China; ^3^ Joint School of National University of Singapore and Tianjin University, International Campus of Tianjin University, Fuzhou, China

**Keywords:** polythiophenes, non-fullerene organic solar cells, molecular interaction, fluorination, miscibility, film morphology

## Abstract

Owing to the advantages of low synthetic cost and high scalability of synthesis, polythiophene and its derivatives (PTs) have been of interest in the community of organic photovoltaics (OPVs). Nevertheless, the typical efficiency of PT based photovoltaic devices reported so far is much lower than those of the prevailing push-pull type conjugated polymer donors. Recent studies have underscored that the excessively low miscibility between PT and nonfullerene acceptor is the major reason accounting for the unfavorable active layer morphology and the inferior performance of OPVs based on a well-known PT, namely PDCBT-Cl and a non-halogenated nonfullerene acceptor IDIC. How to manipulate the miscibility between PT and acceptor molecule is important for further improving the device efficiency of this class of potentially low-cost blend systems. In this study, we introduced different numbers of F atoms to the end groups of IDIC to tune the intermolecular interaction of the hypo-miscible blend system (PDCBT-Cl:IDIC). Based on calorimetric, microscopic, and scattering characterizations, a clear relationship between the number of F atoms, miscibility, and device performance was established. With the increased number of F atoms in IDIC, the resulting acceptors exhibited enhanced miscibility with PDCBT-Cl, and the domain sizes of the blend films were reduced substantially. As a result, distinctively different photovoltaic performances were achieved for these blend systems. This study demonstrates that varying the number of F atoms in the acceptors is a feasible way to manipulate the molecular interaction and the film morphology toward high-performance polythiophene:nonfullerene based OPVs.

## Introduction

In the field of organic solar cells (OSCs), polythiophene and its derivatives have obtained extensive attention due to their low cost and easy accessibility for commercial applications (Mehmood et al., 2016; Wang Q. et al., 2020). Much progress has been acquired in recent years profited from the discovery of a series of novel PTs and nonfullerene acceptors (NFAs) (Liang et al., 2019; Xu et al., 2019; He et al., 2020; Tang et al., 2020; Wang Q. et al., 2020; Ren et al., 2021). In 2016, Hou et al. synthesized a new PT derivative named PDCBT by incorporating electron-withdrawing ester groups into the side chains. When paired with ITIC, a high open-circuit voltage (*V*_oc_) value of 0.94 V and over 10% efficiency were achieved (Qin et al., 2016). Our group further incorporated chlorine atoms into backbones and synthesized the polymer PDCBT-Cl (Wang et al., 2019). An improved efficiency of over 12% was obtained by matching with ITIC-Th1. Moreover, the relatively large-scale and low-cost synthetic methods of the monomers were developed. As reported so far, the carboxylate-substituted PTs deliver the record efficiency in PT:NFA-based blends and exhibit great potential for further applications.

**FIGURE 1:**
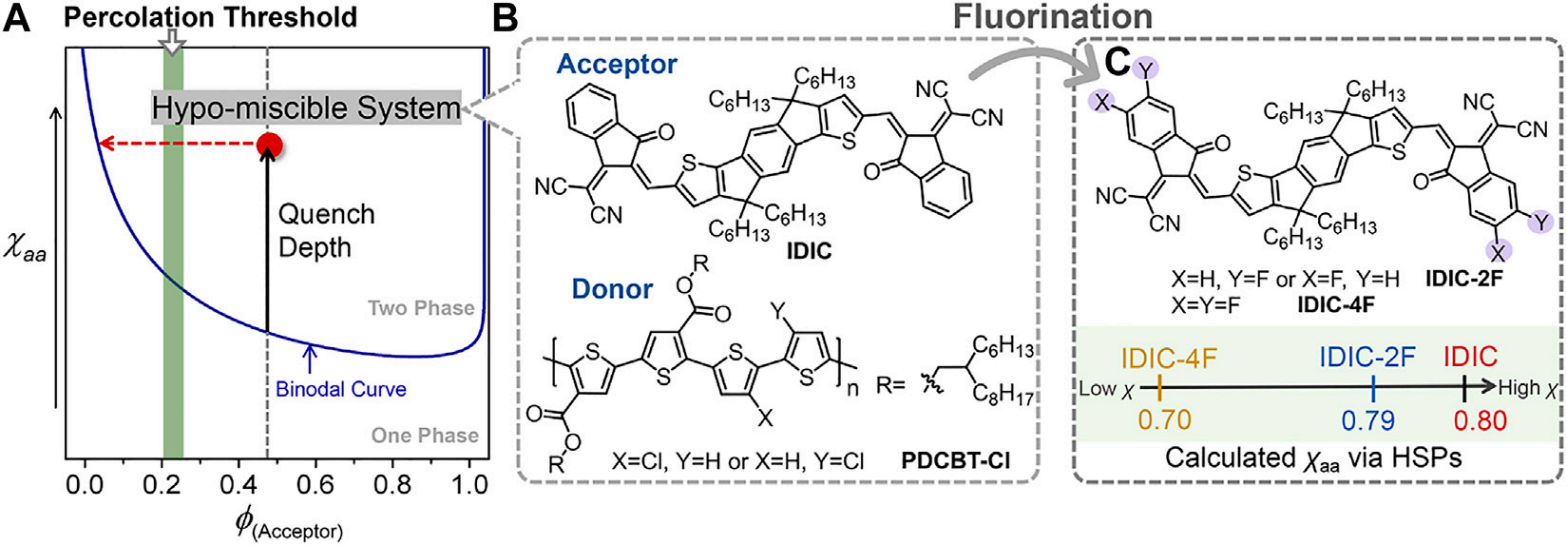
**(A)** Illustration of the Flory–Huggins interaction parameter (*χ*)-volume composition of acceptor (*ϕ*) phase diagram for the hypo-miscible system. **(B)** Chemical structures of IDIC and PDCBT-Cl. **(C)** Chemical structures of IDIC-2F and IDIC-4F, and the illustration of the calculated *χ* according to the Hansen Solubility Parameters.

Although considerable progress has been made, the efficiency of PT-based OSCs still significantly lags behind that of the devices based on push-pull type polymer donors (Zhang et al., 2016; Liu et al., 2018; Wang et al., 2019; Wu et al., 2020; Wu et al., 2021; Zhang et al., 2021). The inferior performance is mainly due to the dissatisfactory morphology of the active layer leading to the increased charge recombination and impeditive charge transport. The final morphology was determined by the thermodynamic molecular interaction and kinetic factors cooperatively during the film-forming process (Gao et al., 2016; Ye et al., 2018b; Gao et al., 2019; Wang Z. et al., 2021). Previous studies have shown that miscibility manipulation of the blending materials could be served as an effective way for performance improvements (Ye et al., 2019; Wang Z. et al., 2020; Yi et al., 2020). Our recent work further demonstrated that the miscibility of PT:NFA systems could be precisely manipulated by molecular structure regulations. We synthesized a series of PT derivatives by incorporating different contents of siloxane-terminated units into the side chains of PDCBT-Cl, a representative of the most recent generation of photovoltaic polythiophenes (Wang Q. et al., 2021). The miscibility between PT and ITIC-Th1 reduced as the content of siloxane side chains increased, resulting in more serious phase separation. As a consequence, the devices based on PDCBT-Cl-Si5:ITIC-Th1 blends delivered the highest efficiency of 12.85%. Further on, the structures of acceptors also exhibit significant impacts on mixing thermodynamic behaviors of the blends. In terms of this issue, we systematically investigated the relationship between molecular structures, miscibility, morphology and performance based on PDCBT-Cl and five classical NFAs (Liang et al., 2020). Among them, PDCBT-Cl and ITIC-Th1 displayed the proper miscibility and relatively high molecular ordering, thus delivering the highest efficiency. In contrast, PDCBT-Cl exhibited the hypo miscibility with the non-halogenated NFA, named IDIC, which provided the large driving force to form the serious phase separation (Figure 1). Eventually, inferior performance and device stability were obtained for the PDCBT-Cl:IDIC blend despite its high crystallinity. How to further manipulate the miscibility of these PT and NFA blends precisely is pivotal. In particular, developing simple and universal design rules is profoundly desired for further prompting the performance of these low-cost systems.

Currently, end group modification is a feasible and effective way for most of NFAs to adjust the photophysical properties and molecular stacking behaviors (Li et al., 2016; Suman and Singh, 2019). Fluorine atoms have been extensively incorporated in terminals due to the merits of strong electrophilic, large polarity, and negligible steric hindrance (Dai et al., 2017; Zhao et al., 2017; Liang et al., 2018; Chen et al., 2021). Moreover, our previous study indicated that introducing fluorine atoms in terminals would likely have a notable impact on molecular interactions (Liang et al., 2020). Based on the above considerations, introducing F atoms in terminals of IDIC might be a valid and feasible way to improve the miscibility with PDCBT-Cl and further prompt the performance. To predict the potential impact of fluorine atoms on the molecular interaction, we firstly performed a quick calculation of amorphous-amorphous interaction parameter (*χ*_*aa*_) with PDCBT-Cl and IDIC-xF according to the Hansen solubility parameters (HSPs) as illustrated in Eq. 1. The HSPs can be estimated easily from the functional group additive methods based on the molecular structures (Leman et al., 2015; Gao et al., 2020; Zhang et al., 2020).

$$\chi_{aa}=\alpha \frac{V_{s}}{RT}\left({\left(\delta_{Dp}-\delta_{Ds}\right)}^{2}+\frac{1}{4}{\left(\delta_{Pp}-\delta_{Ps}\right)}^{2}+\frac{1}{4}{\left(\delta_{Hp}-\delta_{Hs}\right)}^{2}\right)$$ (1)

where *α* is a correction term and tends to 0.5 in polymer (*p*):small molecule (*s*) systems. *V*_s_ refers to the molar volume which is the geometric mean of polymer and small molecule. *R* is the ideal gas constant and *T* is in Fahrenheit. The *δ*_P_, *δ*_D_ and *δ*_H_ in parentheses represent the dispersive interactions, polar interactions and hydrogen bonding interactions, respectively. The detailed data are summarized in Figure 1C and Supplementary Table S1. Compared to the PDCBT-Cl, the acceptors displayed higher *δ*_D_ and *δ*_P_ values but lower *δ*_H_ values which were mainly caused by the differences of polar groups. The *χ*_aa_ values were calculated to be 0.80, 0.79 and 0.70 for IDIC, IDIC-2F and IDIC-4F based blends, respectively. The lower values of *χ*_aa_ represented the better mixing of the components and the results indicated a positive impact of F atoms on miscibility.

Motivated by the above analysis, herein we incorporated mono-fluorinated and double-fluorinated terminals of IDIC, named IDIC-2F and IDIC-4F, respectively, as model systems to test a hypothesis that fluorination of acceptors promotes the miscibility of the hypo-miscible blend system, PDCBT-Cl:IDIC (Figure 1C). With the assistance of theoretical calculation based on HSPs and calorimetric characterizations, a clear and consistent relation of F atoms and miscibility was found. The miscibility between PDCBT-Cl and acceptors was promoted gradually with the increased number of F atoms in terminals. Derived from the proper miscibility of PDCBT-Cl and IDIC-4F, fiber-like morphology was achieved, which leads to the best device PCE along with the intensity maximum of photo response over 70%, much higher than that of the reference system (PDCBT-Cl:IDIC) (∼60%). Moreover, significantly increased device stability could also be observed for the PDCBT-Cl:IDIC-4F system. These results demonstrate that introducing F atoms in end groups of acceptors is a feasible and effective way to finely tune the molecular interaction especially for the hypo-miscible systems and further prompt the increase in the performance of PT:NFA-based photovoltaic devices.

## Materials and Methods

### Materials

The donor material PDCBT-Cl (*M*_n_ = 18.7 kg/mol, PDI = 1.73) used in this work was synthesized according to our previous report (Wang et al., 2019). All acceptors (IDIC, IDIC-2F and IDIC-4F) were synthesized by Knoevenagel condensation as previously reported (Lin et al., 2016). PDINO and anhydrous chloroform were purchased from Derthon Optoelectronic Materials Science Technology Co. Ltd. and Sigma-Aldrich, respectively, and used without further purification.

### Characterization

The UV-vis-NIR absorption spectra and the film cyclic voltammograms (CV) were measured by a Shimadzu UV-3600 Plus spectrometer and CHI6600 electrochemical analyzer, respectively. Differential scanning calorimetry (DSC) curves were tested on a Q25 (TA instruments) differential scanning calorimeter. The whole tests were conducted under the nitrogen atmosphere with heating and cooling rates of 10°C/min. The melting point of various curves was identified as the endset point of the melting peak from the second heating curves. Nanoscale morphology of the samples was characterized by a MutiMode 8 atomic force microscopy (AFM, Bruker) in tapping mode. Transmission electron microscopy (TEM) images were conducted on a JEM-2100PLUS electron microscopy (JEOL) with an accelerating voltage of 200 kV. Grazing incidence wide-angle X-ray scattering (GIWAXS) experiments were carried out in Shanghai Synchrotron Radiation Facility (SSRF), at beamline BL14B1 with an incidence angle of ∼0.2° for complete penetration of X-ray into the films. The X-ray wavelength was 1.24 Å and the beam center along with the sample-to-detector distance were calibrated with LaB_6_.

### Fabrication and Characterization of Photovoltaic Devices

All OSC devices were fabricated with a normal structure of ITO/PEDOT:PSS (35 nm)/PDCBT-Cl:IDIC-xF/PDINO (20 nm)/Al (100 nm). The active layer materials with the D:A ratio of 1:1 and total concentration of 18 mg/ml were dissolved in anhydrous chloroform at least 3 h before spin coating. First, the cleaned ITO glass substrates were processed with UV ozone for 25 min. Then the PEDOT:PSS layer was spin coated on substrates at 4,000 rpm for 20 s, followed by baking at 140°C for 20 min and then being transferred into the glove box. The active layer was spin coated on the surface at 2,000 rpm/min with a thickness of about 100 nm. The film thickness was tested by Dektak150 profilometer (Bruker). Then the films were annealed under the tetrahydrofuran (THF) vapor atmosphere for various time. After that, the solution of PDINO dissolved in methanol was deposited on the surface, and then the aluminum electrode was thermally evaporated under a pressure of <1.5 × 10^−4^ Pa. The effective area of each device is 4 mm^2^ calibrated by the metal mask for solar cells. The J-V curves were tested by Keithley 2400 source meter under the simulated solar light illumination of AM 1.5 G illumination at 100 mW/cm^2^ provided by an AAA solar simulator (Enli Tech, Taiwan). The light intensity was calibrated with a standard photovoltaic cell equipped with a KG5 filter in the glove box. The external quantum efficiency (EQE) spectra were acquired using a solar cell spectral response measurement system (QE-R, Enli Technology Co., Ltd.). The devices were stored in the glove box under dark for stability measurement.

## Results and Discussion

### Photophysical and Electrochemical Properties

**FIGURE 2:**
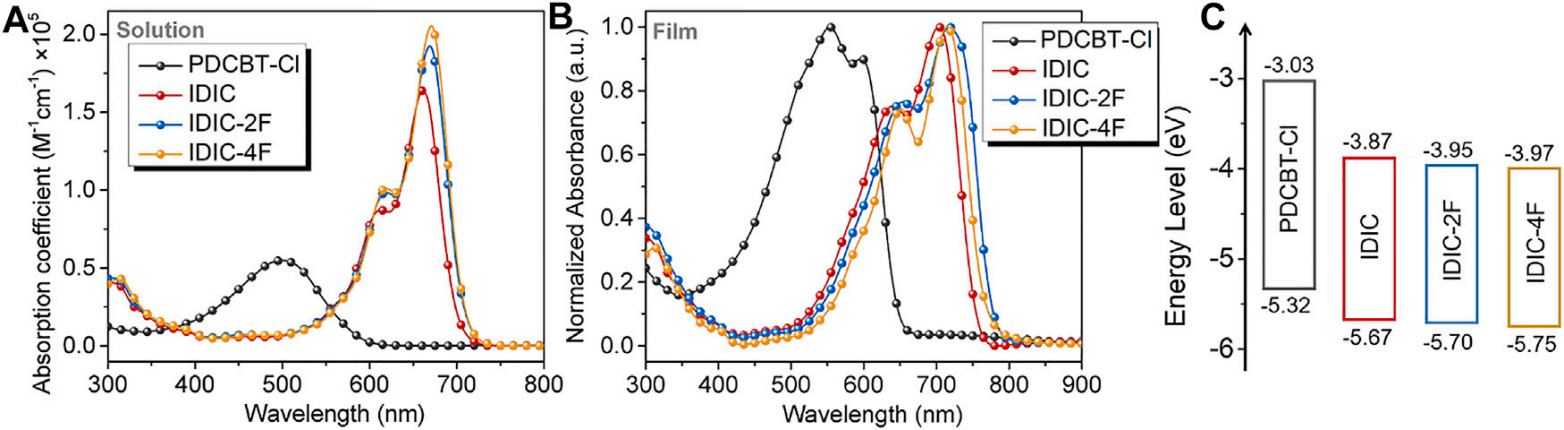
**(A)** Solution (dissolved in chloroform with the concentration of 10^−5^ mol/L) and **(B)** film absorption spectra of PDCBT-Cl and IDIC-xF. **(C)** The schematic energy levels of PDCBT-Cl and IDIC-xF.

The basic optical and electrochemical properties of the acceptors were firstly measured. The solution and thin-film absorption spectra are displayed in Figure 2 and corresponding data are summarized in Supplementary Table S2. In solution, the absorption maximums $\left(\lambda_{\max}^{sol}\right)$ of the acceptors displayed a slight redshift from 661 to 671 nm with the increased number of fluorine atoms in terminals. In addition, higher absorption coefficient was obtained with the enhancement of fluorination which could be ascribed to the increase of intramolecular interaction. From solution to film, all acceptors showed an obvious red shift of the absorption maximum and more obvious vibronic absorption peaks could be observed implying an increased intermolecular interaction. The corresponding optical bandgaps estimated from the onset of absorption edge were 1.63, 1.58 and 1.59 eV for IDIC, IDIC-2F and IDIC-4F, respectively. Moreover, all acceptors displayed the complementary absorption spectra with PDCBT-Cl. The thin-film cyclic voltammograms of acceptors were recorded to calculate the energy levels of materials and the potential was calibrated by ferrocene/ferrocenium (Fc/Fc^+^). The lowest unoccupied molecular orbital (LUMO) and highest occupied molecular orbital (HOMO) energy levels were determined according to the onset of the first reduction and oxidation peaks. As shown in Figure 2C and Supplementary Figure S1, the LUMO and HOMO levels were −3.87 eV/−5.67 eV, −3.95 eV/−5.70 eV and −3.97 eV/−5.75 eV for IDIC, IDIC-2F and IDIC-4F, respectively. The deeper LUMO and HOMO energy levels were acquired due to the introduction of F atoms and the trend of electrochemical gaps was consistent with that of the optical bandgaps.

### Photovoltaic Performance of PDCBT-Cl:IDIC-xF Blends

**FIGURE 3:**
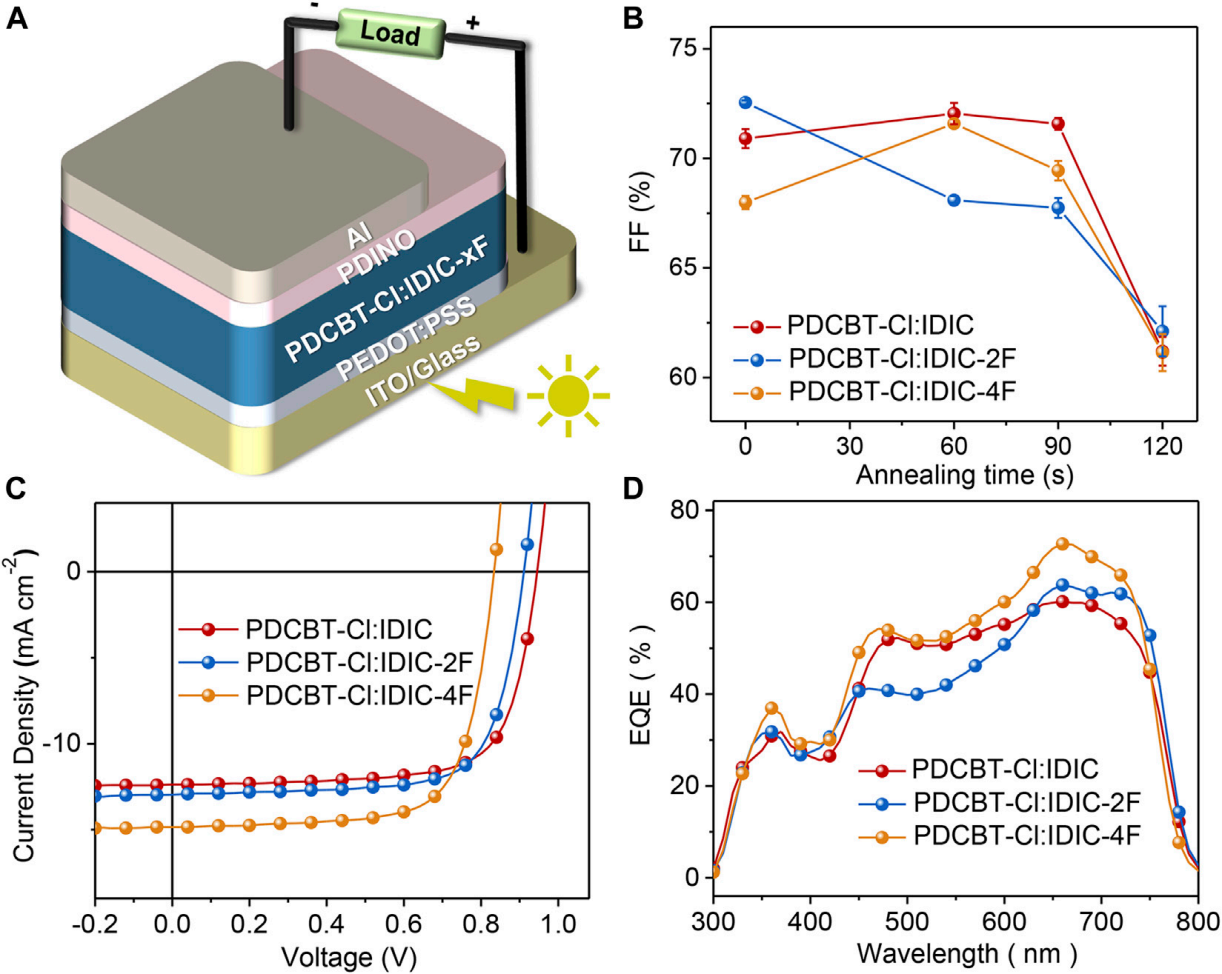
**(A)** Device architecture of OSCs based on PDCBT-Cl:IDIC-xF blends **(B)** Plots of FF as a function of solvent vapor annealing time **(C)** J-V characteristics and **(D)** EQE curves of OSCs under the optimized conditions.

To gain insight into the influence of fluorination on photovoltaic performance, conventional solar cell devices were fabricated with the architecture of ITO/PEDOT:PSS/PDCBT-Cl:IDIC-xF/PDINO/Al. The details of the device fabrication were described specifically in the section *Materials and Methods*. Solvent vapor annealing (SVA) with THF was utilized to further optimize the device performance. The variation of fill factor (FF) as a function of annealing time is illustrated in Figure 3B and the variations of the short-circuit current density (*J*_sc_) and PCE are given in Supplementary Figure S2. It could be seen that the device efficiencies of IDIC and IDIC-2F-based blends were decreased with the prolongation of the SVA process. The optimized performance was acquired of as-cast films with the PCE of 8.60 and 8.58% for IDIC and IDIC-2F-based systems, respectively. While for PDCBT-Cl:IDIC-4F-based blends, the efficiency was firstly improved and then reduced with the increase of SVA time. The best PCE of 9.02% was obtained after annealing for 60 s. The corresponding parameters are listed in Supplementary Tables S3, S4. J-V curves of the optimized devices are displayed in Figure 3C. The best performing devices delivered the open-circuit voltage (*V*_oc_) of 0.95, 0.91 and 0.83 V for IDIC, IDIC-2F and IDIC-4F-based blends, respectively, which could be well explained by the differences of LUMO levels of the acceptors. Compared to the reference system PDCBT-Cl:IDIC with FF of 71.5% and *J*_sc_ of 12.7 mA cm^−2^, the blends based on fluorinated acceptors exhibited the apparently improved FF and *J*_sc_ values of 72.6%, 13.2 mA cm^−2^ for IDIC-2F and 72.5%, 15.0 mA cm^−2^ for IDIC-4F, respectively.

To better understand the performance differences, the relevant EQE curves were acquired as illustrated in Figure 3D and the calculated *J*_sc_ values were in accordance with the values extracted from J-V curves within 5% mismatch. All three blends exhibited similar photo response range from 300 to 800 nm but various response intensities. Among them, PDCBT-Cl:IDIC-4F blend delivered the highest EQE response with the peak value of ∼73% at 665 nm, followed by PDCBT-Cl:IDIC-2F with the maximum of ∼64%, while just approach 60% for PDCBT-Cl:IDIC system. The differences of device performance could be revealed effectively in the variation of morphology which we will discuss in the following with particular emphasis on the mutual effects between the molecular structure, miscibility, morphology, and performance.

### Molecular Ordering and Phase Separation of PDCBT-Cl:IDIC-xF Blends

**FIGURE 4:**
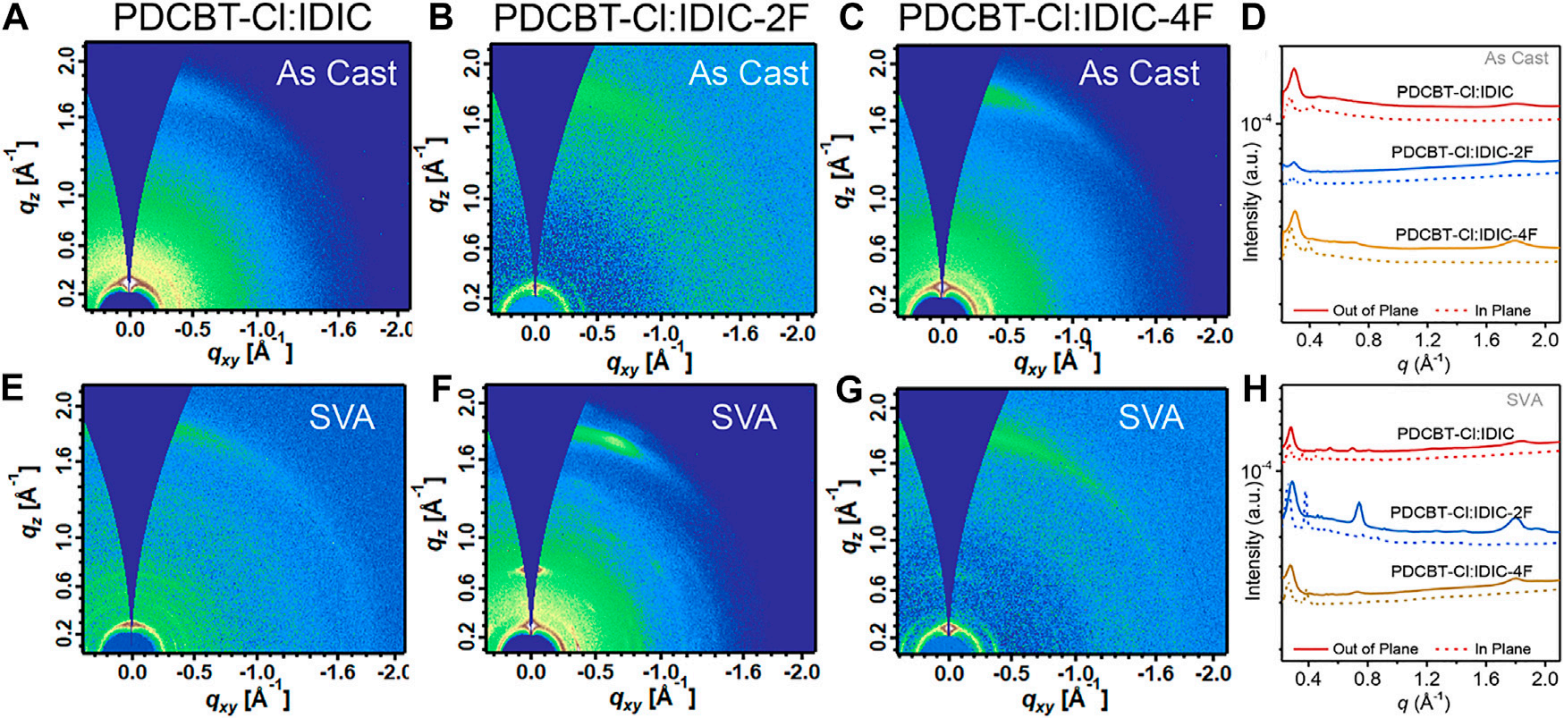
2D GIWAXS patterns for **(A)** IDIC **(B)** IDIC-2F **(C)** IDIC-4F-based blends of the fresh films and **(E)** IDIC **(F)** IDIC-2F **(G)** IDIC-4F-based blends with solvent vapor annealing for 60s **(D)** 1D profiles derived from **(A)** to **(C)** and **(H)** derived from **(E)** to **(G)**.

Film ordering and morphology have vital influences on solar cell performance. Therefore, to probe the information of molecular ordering and crystal texture of the blends, GIWAXS measurements were carried out at SSRF. The GIWAXS patterns along with the corresponding 1D profiles of PDCBT-Cl:IDIC, PDCBT-Cl:IDIC-2F and PDCBT-Cl:IDIC-4F blend films without and with SVA treatments are illustrated in Figure 4. The relevant peak-fitting results and detailed data are given in Supplementary Figure S3 and Supplementary Table S5. For the fresh films, all three blends exhibited the (100) diffraction peaks at 0.29 Å^−1^ with the corresponding *d*-spacing of 21.6 Å in *q*_z_ direction which can be ascribed to the lamellar packing of PDCBT-Cl (Wang et al., 2019). In addition, (010) diffraction peaks arising from π-π stacking were also observed at 1.79 Å^−1^ along the *q*_z_ directions for PDCBT-Cl:IDIC-4F blend film, while IDIC and IDIC-2F-based blend films exhibited the (010) peak at 1.80 Å^-1^ with a slightly tighter *d*-spacing of 3.49 Å. The corresponding π-π coherence lengths (*L*_c_) calculated according to the Scherrer Equation were 31.4, 31.4 and 33.3 Å for IDIC, IDIC-2F and IDIC-4F-based blend films, respectively. After SVA treatments, more pronounced (010) diffraction peaks were observed with the *L*_c_ values increased to 40.4, 51.4, 47.1 Å for IDIC, IDIC-2F and IDIC-4F-based blends, indicating an improved molecular ordering. The paracrystallinity parameter (*g*) that describes the standard deviation of local static lattice fluctuations was estimated according to the approximate formula (Noriega et al., 2013): $g\approx \left(1/2\pi \right)\sqrt{\Delta_{q}d_{hkl}}$, where $\Delta_{q}$ and *d*_hkl_ represents the full width at half maximum (FWHM) and *d*-spacing of the relevant diffraction peak, respectively. The respective *g* values were calculated to be 11.0, 9.9 and 10.3% for IDIC, IDIC-2F and IDIC-4F-based blend films. The more compact stacking with the lower *g* value could be considered as one of the reasons for the efficiency improvement of IDIC-4F-based system. Particularly, multiple diffraction features could be observed at the *q* range of 0.3 ∼ 0.9 Å for IDIC and IDIC-2F-based blends, indicating the crystallization of acceptors after annealing especially for PDCBT-Cl:IDIC-2F system which displayed the smallest *g* value and the highest *L*_c_.

**FIGURE 5:**
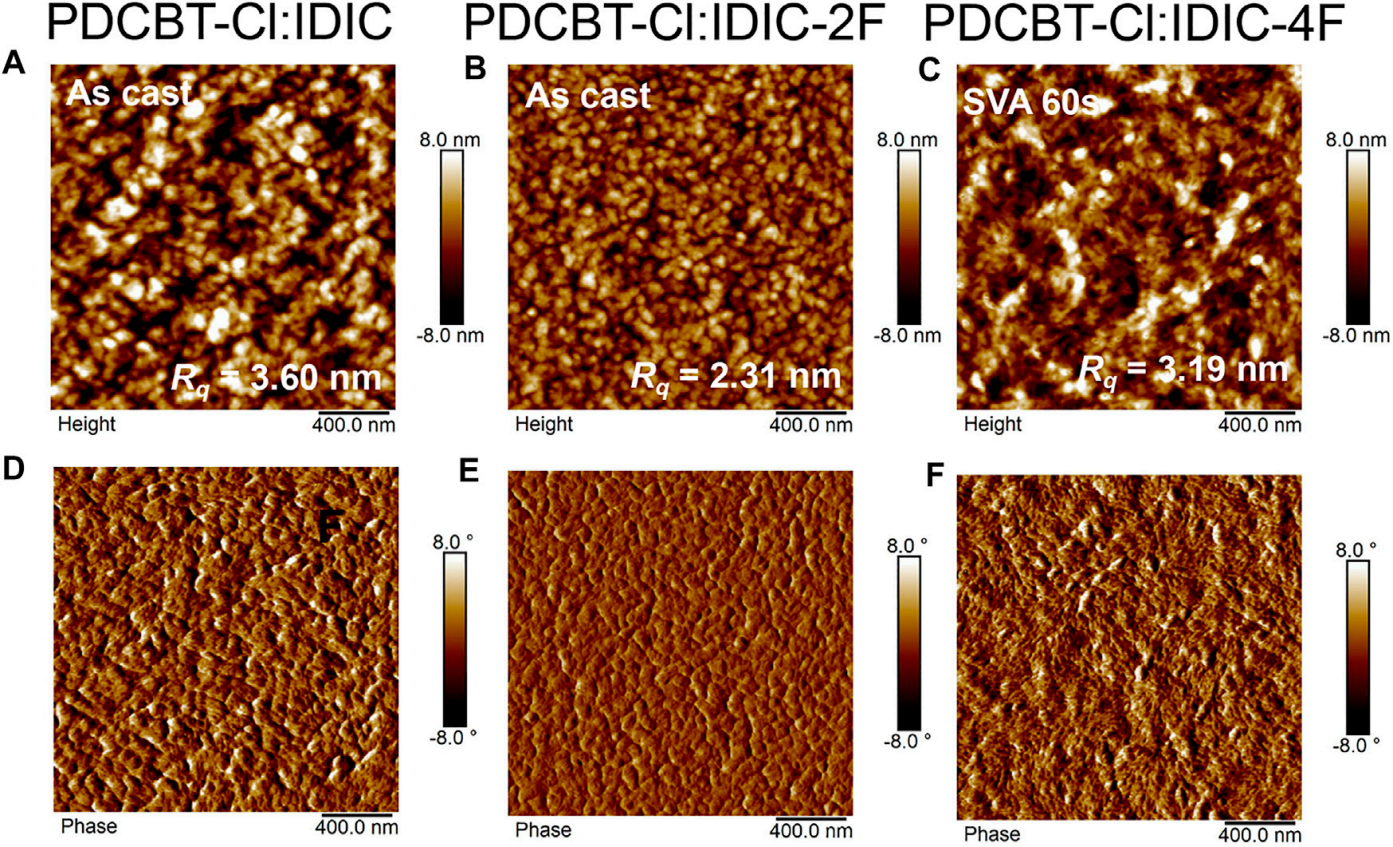
AFM height images **(A–C)** and phase images **(D–F)** for PDCBT-Cl:IDIC **(A,D)**, PDCBT-Cl:IDIC-2F **(B,E)** and PDCBT-Cl:IDIC-4F **(C,F)**-based blends under the optimized conditions.

To reveal the scale of phase separation of these three blends, the film morphology was investigated by tapping-mode AFM. As illustrated in Figure 5, the optimized IDIC and IDIC-2F-based blend films, i.e. the as-cast blend film, delivered the granular-like structure with large phase separation, indicating the serious aggregations of the materials. By contrast, the morphology of the as-cast PDCBT-Cl:IDIC-4F film was more uniform (Supplementary Figure S4). After SVA treatments for 60 s, the PDCBT-Cl:IDIC-4F film exhibited more obvious fibrous morphology with proper phase separation (Figure 5), which was favorable for charge transport, supporting the high *J*_sc_ and PCE values. However, after SVA treatments of IDIC and IDIC-2F-based films, visibly increased phase separation and molecular aggregation were observed as shown in Supplementary Figure S5 with the root-mean-square roughness (*R*_*q*_) values reaching up to 11.8 and 7.5 nm for IDIC and IDIC-2F-based blends, respectively. TEM images exhibited similar morphological characteristics under SVA conditions. Particularly for IDIC-2F-based blends, the sheet-like morphology was observed in both AFM and TEM images. These domains could be considered as the IDIC-2F aggregations supported by GIWAXS results. The large phase separation and serious molecular aggregations could be responsible for the decreased FF values and device performance.

### Molecular Interaction of PDCBT-Cl:IDIC-xF Blends

**FIGURE 6:**
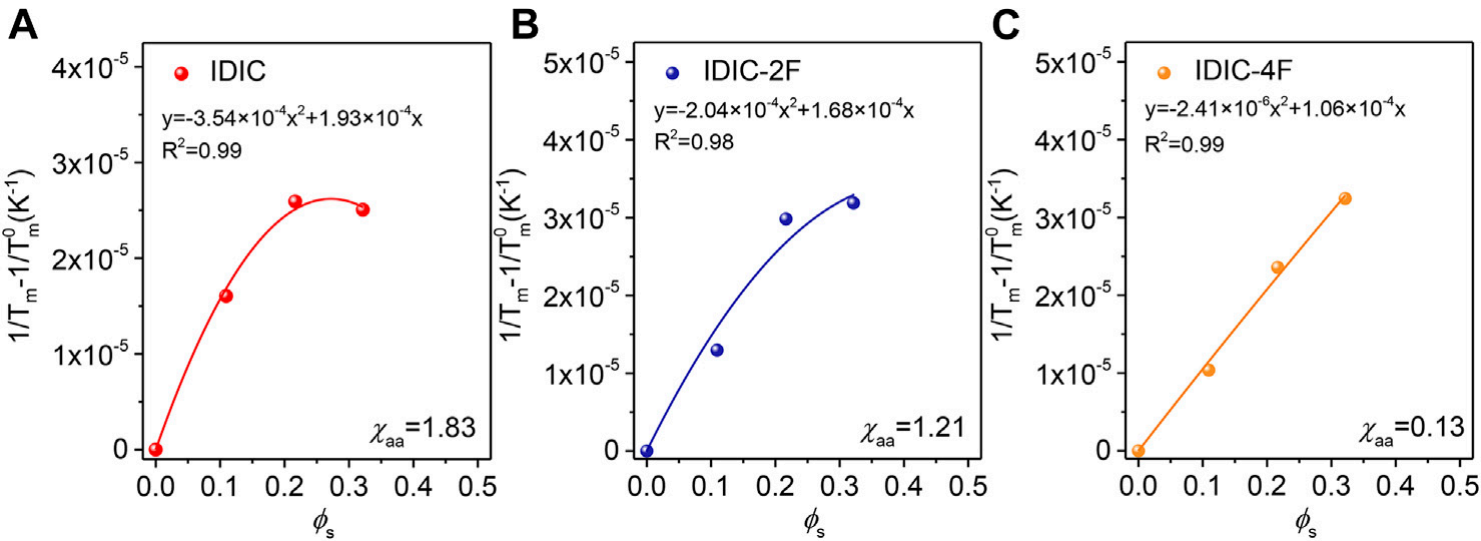
Measurements of melting point depression to estimate the miscibility of PDCBT-Cl:IDIC-xF blends as a function of the volume fraction of acceptors **(A)** IDIC **(B)** IDIC-2F **(C)** IDIC-4F.

The morphological characteristics are largely dominated by the thermodynamic factors of the blends (White et al., 2012; Ye et al., 2018b). Consequently, the molecular interactions between PDCBT-Cl and IDIC-xF were further verified through DSC measurements according to the theory of melting point depression (Nishi and Wang, 1975). The melting points of pure PDCBT-Cl and the blends with various blending ratios were obtained as shown in Supplementary Figure S6. With the amounts of acceptors increasing, the melting temperature of PDCBT-Cl was decreased gradually and IDIC-4F-based blends exhibited the steep and continuous descent as illustrated in Supplemntary Figure S7. This phenomenon indicated the largest disruption for the ordered arrangement of PDCBT-Cl crystals when blending with IDIC-4F. The specific values of *χ*_aa_ could be obtained according to Eq. 2 (Kozub et al., 2011).

$$\frac{1}{T_{m}}-\frac{1}{T_{m}^{0}}=\frac{R}{\Delta H_{f}}\frac{v_{m}}{v_{s}}\left(\phi_{s}-\chi_{aa}\phi_{s}^{2}\right)$$ (2)

where $T_{m}^{0}$ and *T*_m_ are the melting points of the pure PDCBT-Cl and in blends, respectively. $\Delta H_{f}$ refers to the melting enthalpy of the neat PDCBT-Cl. $v_{m}$ and $v_{s}$ identify the molar volume of the constitutional unit of PDCBT-Cl and acceptors. *ϕ*_*s*_ is the volume fraction of acceptors, and *R* represents the ideal gas constant. By fitting the melting point data into Eq. 2, the *χ*_aa_ value of PDCBT-Cl:IDIC blend was calculated to be 1.83 which was similar to our pervious report and the numerical difference could be ascribed to the variations of molecular weight and polydispersity index of PDCBT-Cl (Liang et al., 2020). The *χ*_aa_ values were found to be 1.21 and 0.13 for IDIC-2F and IDIC-4F-based blends, respectively (Figure 6). Accordingly, the *χ*_aa_ obtained from the DSC and HSP method displayed a consistent trend, i.e. PDCBT-Cl:IDIC > PDCBT-Cl:IDIC-2F > PDCBT-Cl:IDIC-4F. Consequently, it could be firmly demonstrated that the enhanced molecular interaction could be achieved with the increasing fluorination in end groups. Therefore, the incorporation of fluorine atoms could be a simple but effective way to manipulate the blend miscibility precisely. The relations between miscibility and film nanostructure will be discussed in further detail below.

### The Effect of Fluorination on Molecular Interaction and Performance

**FIGURE 7:**
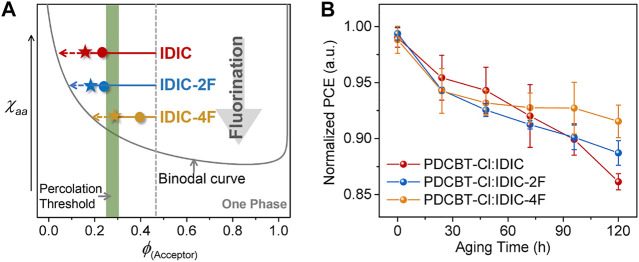
**(A)** Illustration of the amorphous *χ*_aa_ *-ϕ*_s_ phase diagram based on PDCBT-Cl:IDIC-xF blends. The dots and pentacles represent the states of the as-cast films and SVA (60s)-treated films, respectively. The arrows indicate the evolution of the compositions for the mixed domains during the SVA and aging process. **(B)** Normalized PCE vs. continuous aging time for PDCBT-Cl:IDIC-xF based devices. The error bars represent standard deviations of at least eight devices for each pair.

Based upon the above characterizations, we can more integrally analyze the impact of fluorination on molecular interaction and performance. Primarily, the miscibility between polymer and NFAs was gradually decreased with the increased number of F atoms in end groups of the acceptors. This variation might likely arise from the fact that the incorporation of fluorine atoms would lower the surface energy of materials and increase the intermolecular interaction caused by the polarity and electronegativity characteristics. Accordingly, the morphological parameters and photovoltaic performance of the blends exhibited apparent differences. For the hypo-miscible system, PDCBT-Cl:IDIC, the oversized phase separation with the less physical interfacial area of donor:acceptor for the as-cast film will induce serious monomolecular recombination and lead to a low *J*_sc_ (12.7 mA cm^−2^). PDCBT-Cl:IDIC-2F with a higher miscibility delivered the smoother surface with reduced domain size in the as-cast film, and thus acquired a higher *J*_sc_ of 13.2 mA cm^−2^. The efficiencies were gradually decreased with the prolongation of SVA treatments for both IDIC and IDIC-2F-based blends, implying that the morphology was less kinetically quenched even for the fresh films being processed with the volatile solvents, which could be mainly ascribed to the hypo-miscibility of the materials. In contrast, for more miscible system PDCBT-Cl:IDIC-4F, the lower efficiencies of the fresh films were obtained compared to that of the SVA-treated films, indicating a deep quenching far away from the percolation threshold for the as-cast films. The champion performance was acquired through the SVA treatments for 60 s, demonstrating that the composition of mixed domains approached the percolation threshold. Combined with the enhanced film crystallinity and more fibrous nanostructure of the blends, the PDCBT-Cl:IDIC-4F-based blends delivered the highest efficiency of over 9% with the much higher *J*_sc_ value of 15.0 mA cm^−2^. Based upon the above analysis, the detailed relations could be delineated and exemplified in the phase diagram as illustrated in Figure 7A.

Beyond an apparent impact on initial performance, the miscibility of donor and acceptor has a significant influence on device stability likewise due to the non-equilibrium state of the blend films (Ye et al., 2018a; Ghasemi et al., 2021). Therefore, the shelf-aging stability of three blends was measured with the unencapsulated devices being stored in an argon-filled glove box at room temperature. As given in Figure 7B, the photovoltaic devices displayed enhanced stability with the improvement of donor:acceptor miscibility. Among them, the PDCBT-Cl:IDIC-4F blends exhibited the best device stability with a degradation loss of less than 10% after 120 h of aging. While for IDIC-based system, a continuous and rapid descent was observed. The decline of efficiencies was primarily related to the decrease of FF as shown in Supplementary Figure S8. According to the established miscibility-function model, the burn-in degradation of the devices was mainly driven by the hypo-miscibility with the inducing of spontaneous demixing (Li et al., 2017; Ye et al., 2018a). Consequently, the above experimental observations indicated that the device performance and aging stability could be simultaneously improved via fluorination in terminals of acceptors.

Currently, fluorination of end groups has been verified as an effective way to control the intermolecular interaction and further boost the device performance by a wide range of studies, which mainly contributed to the extended absorption range, enhanced film crystallinity and improved charge mobility (Dai et al., 2017; Zhang et al., 2017; Zhan et al., 2018; Lai et al., 2020). Hou et al. established a general relationship between molecular interactions and molecular structure by investigating the surface electrostatic potential of the materials. They discovered that the intermolecular interactions of donor and acceptor were enhanced with more fluorine atoms incorporated in acceptors (Xu et al., 2020). In addition, a recent work by S. Swick *et al.* further demonstrated that fluorination of NFAs could prompt face-to-face packing and form the compact stacking by crystal structure analysis, thus leading to the increased performance (Swick et al., 2020). Further on, this work primarily focused on the effect of fluorination on thermodynamic interaction and morphology. The corresponding results indicated that manipulation of fluorination for acceptors would be a simple and viable way to regulate the molecular interaction. A similar consequence was also observed in a study of all-polymer solar cells by Peng and coworkers (Peng et al., 2020). Besides, Hou et al. designed and synthesized a fluorinated PT derivative named P302 in a most recent study, which displayed the much enhanced molecular planarity compared to that of the non-fluorinated counterpart. More significantly, P302 delivered the reduced miscibility with a Y-series derivative, i.e., Y5 and formed a more favorable phase separation, leading to the apparently improved efficiency by a factor of ∼4 (Yang et al., 2021). These results reveal that fluorination plays a crucial role in the performance regulations of many OPV systems. Combining with our experimental results, fluorination of acceptors probably could precisely control the intermolecular interaction for hypo-miscible systems. Low-cost blend systems are expected to be further prompted by precisely adjusting the location and numbers of fluorination of both donor and acceptor molecules.

## Conclusion

In summary, the function of fluorination toward molecular interaction and photovoltaic performance has been studied in detail based on a polythiophene derivative PDCBT-Cl and three NFAs with different numbers of fluorine atoms, namely IDIC, IDIC-2F, IDIC-4F. By means of scattering, calorimetric and microscopic techniques, the relations of molecular structure, morphology and performance were finely established. Primarily, the molecular interaction of hypo-miscible systems can be precisely regulated by tunning the fluorine numbers in the terminals. The donor:acceptor miscibility was gradually decreased with the reduced contents of fluorination, leading to the more serious phase separation caused by the larger driving force. For the blends of IDIC and IDIC-2F, due to the hypo-miscibility with PDCBT-Cl, the morphology was less kinetically quenched even for the as-cast films being spin-coated from the volatile solvent. The optimal efficiencies were obtained for the fresh films with much lower *J*_sc_ values of 12.7 and 13.2 mA cm^−2^ for IDIC and IDIC-2F-based blends, respectively. The large phase separation and apparent aggregations in films could be responsible for the inferior performance. Further incorporating two fluorine atoms, PDCBT-Cl:IDIC-4F delivered the higher miscibility and more fiber-like nanostructure with a proper domain size of the blend film, contributing to the highest *J*_sc_ value of 15.0 mA cm^−2^ and PCE of over 9% after SVA treatments for 60 s. Moreover, the devices based on PDCBT-Cl:IDIC-4F also exhibited apparently enhanced stability compared to that of IDIC and IDIC-2F-based systems. While previous studies mainly elucidate the impact of fluorination on molecular ordering and stacking, this study further reveals the profound effect on thermodynamic behaviors. Simultaneously, this study also provides a feasible means for miscibility tuning toward high-efficiency devices based on polythiophene:acceptor systems.

## Data Availability

The original contributions presented in the study are included in the article/Supplementary Material, further inquiries can be directed to the corresponding authors.
