# Supplementary material for: Fluorination Enables Tunable Molecular Interaction and Photovoltaic Performance in Non-Fullerene Solar Cells Based on Ester-Substituted Polythiophene
Source: Front Chem. 2021 May 10;9:687996. doi: 10.3389/fchem.2021.687996 (PMC8141579; doi:10.3389/fchem.2021.687996)
Supplement: Supplementary file 1 [file DataSheet1.pdf]

Supplementary Material

Fluorination Enables Tunable Molecular Interaction and Photovoltaic Performance in Nonfullerene Solar Cells Based on Ester-Substituted Polythiophene

Ziqi Liang[1], Mengyuan Gao[1], Bo Zhang[1], Junjiang Wu[1], Zhongxiang Peng[1], Miaomiao Li[1*], Long Ye[1]*, and Yanhou Geng[1,2]

[1]School of Materials Science and Engineering and Tianjin Key Laboratory of Molecular Optoelectronic Science, Tianjin University, Tianjin 300072, P. R. China

[2]Joint School of National University of Singapore and Tianjin University, International Campus of Tianjin University, Binhai New City, Fuzhou 350207, China

***Correspondence:**
Corresponding Authors: Long Ye, Miaomiao Li
Email: yelong@tju.edu.cn; miaomiao.li@tju.edu.cn

Table S1. Calculated HSPs and χ values for PDCBT-Cl and IDIC-xF

	δ_D	δ_P	δ_H	V (cm^3/mol)	χ
PDCBT-Cl	17.0	4.3	5.6	831.1	--
IDIC	18.6	6.8	4.2	868.3	0.80
IDIC-2F	18.6	6.6	4.0	878.1	0.79
IDIC-4F	18.5	6.2	3.8	883.0	0.70

Table S2. Optical absorption and electrochemical properties of PDCBT-Cl and IDIC-xF

	λ_{max}^{sol}(nm)	λ_{max}^{film}(nm)	E_g^{opt}(eV)	E_{LUMO} (eV)	E_{HOMO} (eV)
PDCBT-Cl	498	553, 597	1.90	-3.03	-5.32
IDIC	611, 661	638, 703	1.63	-3.87	-5.67
IDIC-2F	618, 669	653, 720	1.58	-3.95	-5.70
IDIC-4F	619, 671	651, 716	1.59	-3.97	-5.75

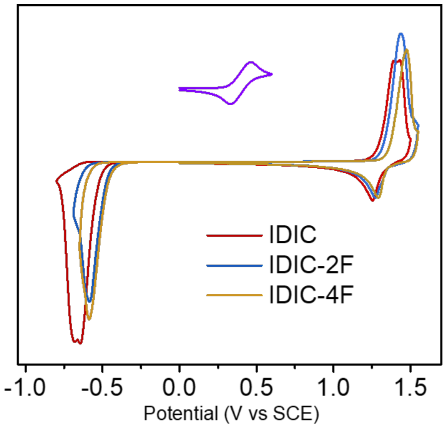

Figure S1. Thin film cyclic voltammograms (CV) curves of IDIC-xF with the scan rate of 0.1 V/s.

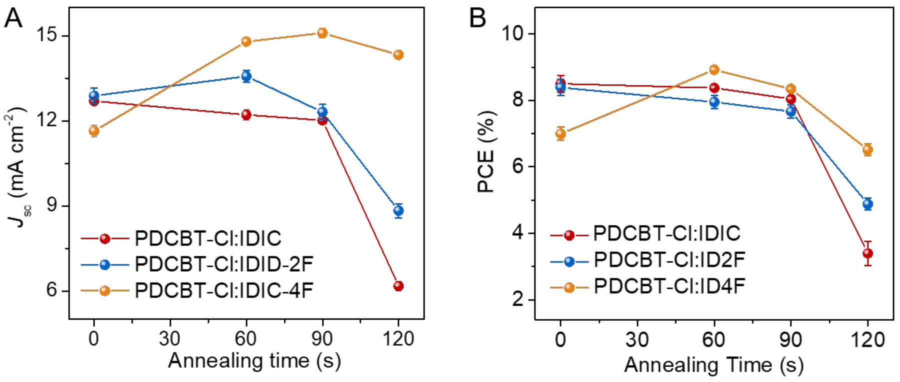

Figure S2. Plots of J_{sc} (A) and PCE (B) as a function of solvent vapor annealing time for PDCBT-Cl:IDIC-xF based blends.

Table S3. Optimized photovoltaic parameters of PDCBT-Cl:IDIC-xF blends

Acceptor	Treatment	V_{oc} (V)	J_{sc} (mA·cm^{-2})	FF (%)	PCE (%)
IDIC	As Cast	0.95±0.01 (0.95)	12.5±0.1 (12.7)	70.9±0.5 (71.5)	8.43±0.15 (8.60)
IDIC-2F	As Cast	0.90±0.01 (0.91)	12.8±0.2 (13.2)	72.3±0.3 (72.6)	8.37±0.20 (8.58)
IDIC-4F	SVA 60s	0.82±0.01 (0.83)	14.7±0.2 (15.0)	71.6±0.6 (72.5)	8.84±0.13 (9.02)

Table S4. Photovoltaic parameters of PDCBT-Cl:IDIC-xF blends with the treatments of SVA for various time.

Acceptor	Treatment	V_{oc} (V)	J_{sc} (mA·cm^{-2})	FF (%)	PCE (%)
IDIC	As Cast	0.95	12.7	71.5	8.60
	SVA 60s	0.95	12.4	72.4	8.47
	SVA 90s	0.94	12.1	71.9	8.12
	SVA 120s	0.94	6.4	61.7	3.54
IDIC-2F	As Cast	0.91	13.2	72.6	8.58
	SVA 60s	0.87	13.9	68.3	8.19
	SVA 90s	0.87	12.5	69.0	7.39
	SVA 120s	0.87	9.1	62.2	4.92

IDIC-4F	As Cast	0.89	11.8	68.3	7.09
	SVA 60s	0.83	15.0	72.5	9.02
	SVA 90s	0.81	15.3	69.4	8.37
	SVA 120s	0.75	14.5	61.8	6.68

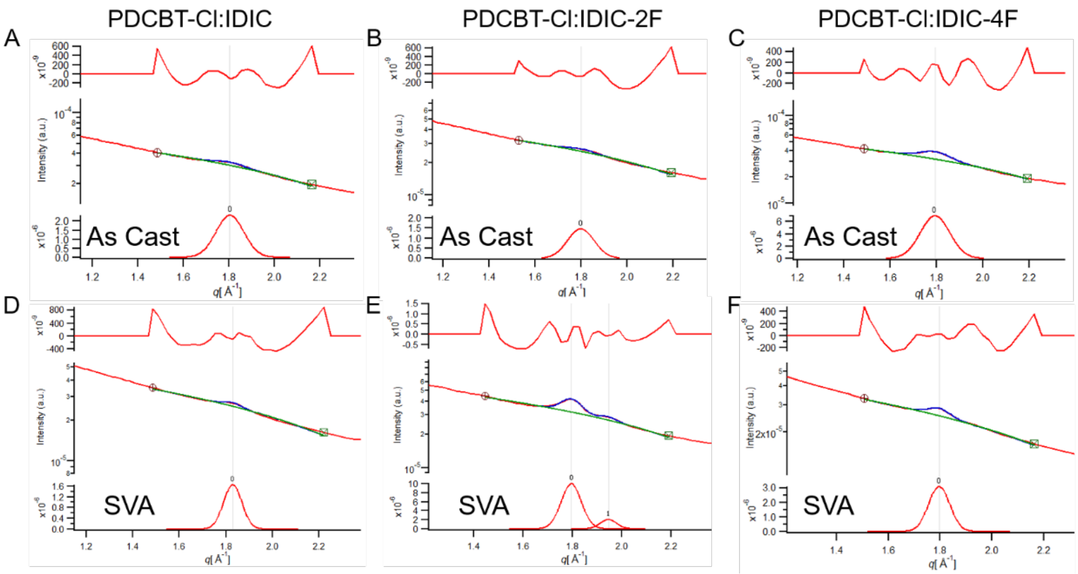

Figure S3. Fitting results of (010) diffraction peaks in out-of-plane direction for PDCBT-Cl:IDIC-xF blends without and with SVA treatments for 60s.

Table S5. Crystalline information of (010) diffraction peaks in q_z direction of PDCBT-Cl:IDIC-xF blends without and with SVA treatments for 60s.

	Treatment	q (Å^{-1})	d-spacing (Å)	FWHM (Å^{-1})	L_c (Å)	g (%)
PDCBT-Cl	As cast	1.73	3.63	0.23	23.8	
PDCBT-Cl:IDIC	As cast	1.80	3.49	0.18	31.4	12.6
	SVA	1.83	3.43	0.14	40.4	11.0
PDCBT-Cl:IDIC-2F	As cast	1.80	3.49	0.18	31.4	12.6
	SVA	1.80	3.49	0.11	51.4	9.9
PDCBT-Cl:IDIC-4F	As cast	1.79	3.51	0.17	33.3	12.3
	SVA	1.79	3.51	0.12	47.1	10.3

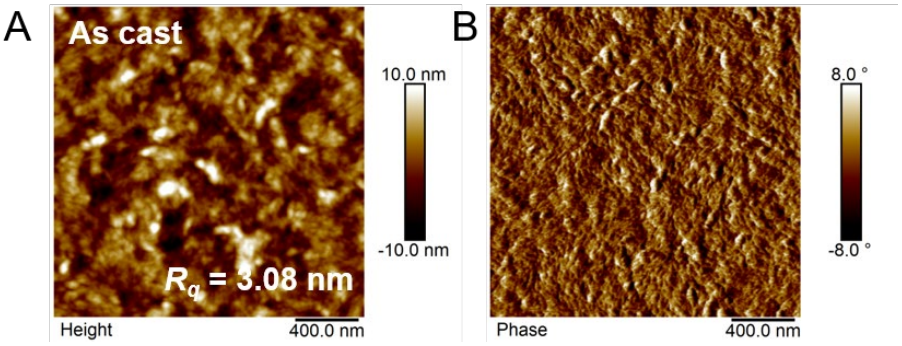

Figure S4. AFM height (A) and phase (B) images for PDCBTC-l:IDIC-4F as-cast films.

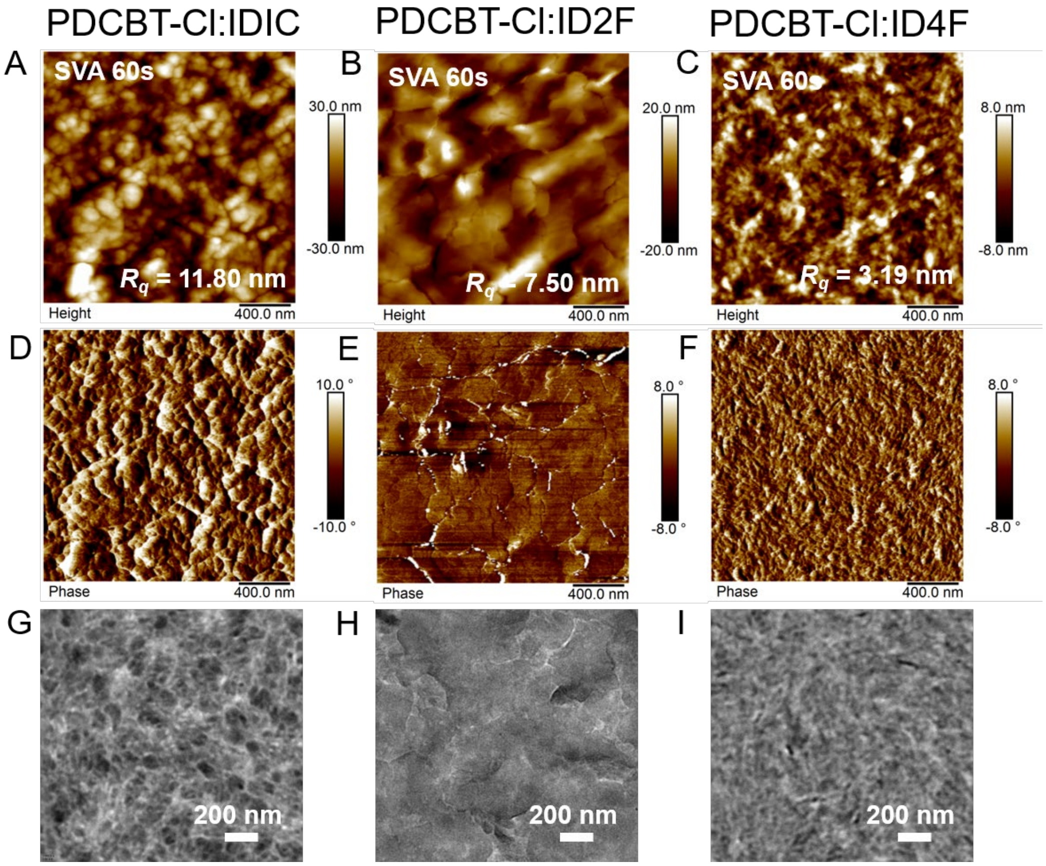

Figure S5. AFM height (A-C) and phase (D-F) images, and TEM images (G-I) for PDCBTC-l:IDIC-xF with SVA treatments for 60s.

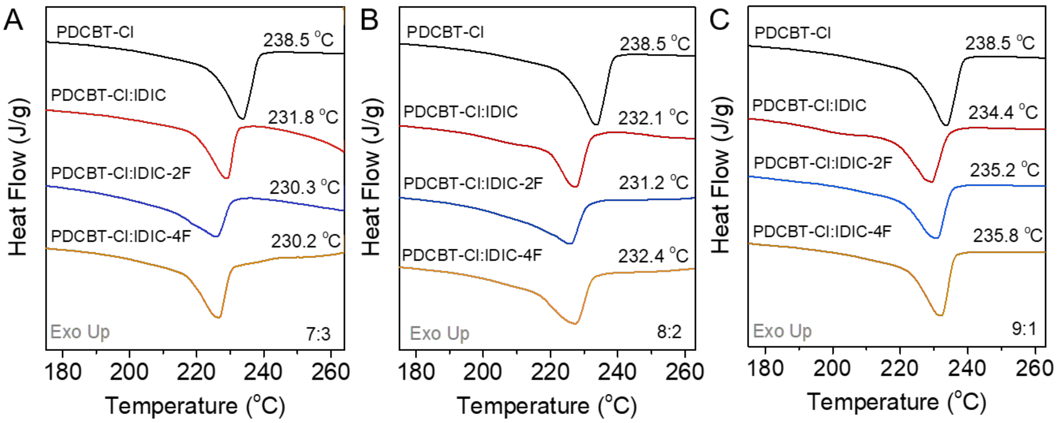

Figure S6. The second cycle of DSC heating curves for pure PDCBT-Cl and PDCBT-Cl:IDIC-xF blends with the different weight ratio of 7:3 (A), 8:2 (B) and 9:1 (C).

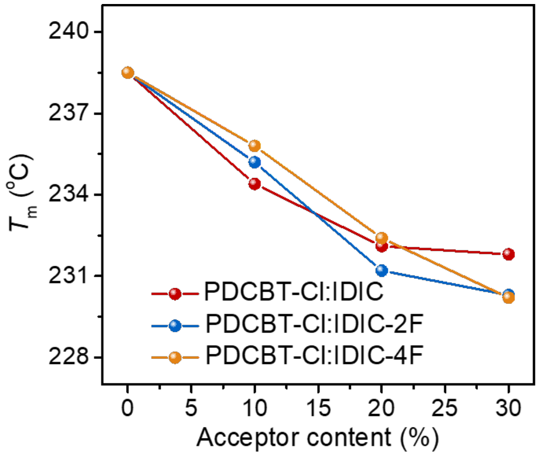

Figure S7. Plots of the melting temperature (T_m) of PDCBT-Cl:IDIC-xF blends with various blending ratios.

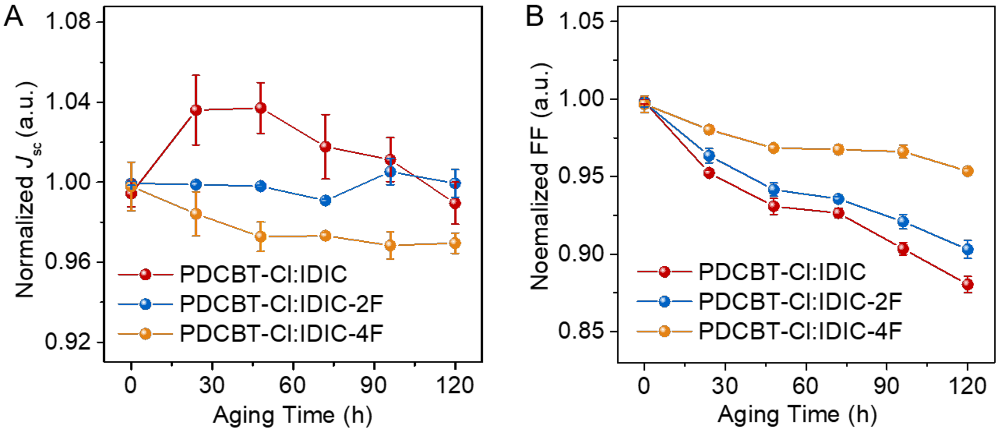

Figure S8. Normalized J_{sc} (A) and FF values (B) versus shelf-aging time for PDCBT-Cl:IDIC-xF based OSCs being stored in the glove box. The error-bars represent the standard deviations for at least six devices.
